# Improving the Signal-to-Noise Ratio of Axial Displacement Measurements of Microspheres Based on Compound Digital Holography Microscopy Combined with the Reconstruction Centering Method

**DOI:** 10.3390/s24092723

**Published:** 2024-04-24

**Authors:** Yanan Zeng, Qihang Guo, Xiaodong Hu, Junsheng Lu, Xiaopan Fan, Haiyun Wu, Xiao Xu, Jun Xie, Rui Ma

**Affiliations:** 1 College of Engineering and Technology, Tianjin Agricultural University, Jinjing Road, Tianjin 300384, China; 2 State Key Laboratory of Precision Measuring Technology and Instruments, Tianjin University, Weijin Road, Tianjin 300072, China; 3 School of Life Sciences, Tiangong University, Tianjin 300387, China; 4 Tianjin Key Laboratory of Intelligent Breeding of Major Crops, Jinjing Road, Tianjin 300392, China

**Keywords:** microsphere, axial displacement measurement, digital holographic microscopy, signal-to-noise ratio, nanometer

## Abstract

In 3D microsphere tracking, unlike in-plane motion that can be measured directly by a microscope, axial displacements are resolved by optical interference or a diffraction model. As a result, the axial results are affected by the environmental noise. The immunity to environmental noise increases with measurement accuracy and the signal-to-noise ratio (SNR). In compound digital holography microscopy (CDHM)-based measurements, precise identification of the tracking marker is critical to ensuring measurement precision. The reconstruction centering method (RCM) was proposed to suppress the drawbacks caused by installation errors and, at the same time, improve the correct identification of the tracking marker. The reconstructed center is considered to be the center of the microsphere, rather than the center of imaging in conventional digital holographic microscopy. This method was verified by simulation of rays tracing through microspheres and axial moving experiments. The axial displacements of silica microspheres with diameters of 5 μm and 10 μm were tested by CDHM in combination with the RCM. As a result, the SNR of the proposed method was improved by around 30%. In addition, the method was successfully applied to axial displacement measurements of overlapped microspheres with a resolution of 2 nm.

## 1. Introduction

As major particles, microspheres are an essential marker in characterization in force- or torsion-dependent molecular processes [1,2], fluid dynamics [3,4], single-molecule force spectroscopy [5], airborne particulate matter research [6,7], etc. They are also a favorable tool to utilize in microscopy for super-resolution imaging [8,9]. Tracking microspheres and quantitatively measuring their three-dimensional motion is vital to revealing the physical or biological principles involved in microsphere experiments. Since the feature size of microspheres is on the micrometer or sub-micrometer scale, optical microscopy is the primary method for measuring the 3D displacement of moving microspheres. The high resolution of displacement at the nanometer scale is a challenging but significant endeavor. Digital holographic microscopy (DHM), which is not a scanning-based method [10], has a nanometer resolution [11]; therefore, it is an approach applicable to dynamic phenomena, including those in microfluidics. Commercial holographic particle characterization instruments (Spheryx, Inc., xSight, New York, NY, USA) yield the microsphere’s in-plane displacement to within a nanometer and its axial position to within 5 nm [12].

Recently, researchers have focused their efforts on two aspects. One is to broaden the application of microsphere measurement, such as the combination of DHM with optical tweezers [13,14], DHM coupled with magnetic tweezers [5], and porous sphere model creation [15,16]. The other one is to optimize the measurement, that is, to discuss the uncertainty of particle tracking [17], to improve efficiency by combining deep learning [18,19], and to reconstruct the 3D particle field by neural network [20], etc.

In fact, the improved accuracy of the underlying measurement has provided a more accurate data basis for applications. The discussion on measurement mechanisms has now developed to the point where researchers are paying more attention to the differences between the practical factors caused by the actual measurement process and the ideal measurement model, such as the reconstruction of tilt surfaces [21] and the elimination of twin images [22].

Improving immunity to noise is a constant subject of progress in measuring technology. Researchers made an effort to suppress noise and improve SNR by adopting light sources with partial coherence [23], recording image-plane holograms [24], using image processing methods [25,26], etc. The immunity to noise increases with measurement precision and signal-to-noise ratio (SNR). As frequency filtering is necessary in DHM, especially for off-axis DHM, the SNR is inevitably sacrificed. The inherent disadvantage limits the instrumentation and application of DHM. To make up for the disadvantage of SNR, those factors that introduce errors should be suppressed. In our previous work, compound digital holographic microscopy (CDHM) was utilized to break through the measuring limitation of the in-focused or nearly in-focused microspheres [27]. However, measuring errors were caused in three aspects. One was optical axis tilt caused by mechanical installation error; the second was the Coma aberration brought by the beam expanding; and the third was the environment noise introduced by stray light, mechanical vibration, CCD white noise, etc. Accurate identification of the tracking marker is the key to suppressing errors, especially for environmental noise. This is due to the fact that the amplitude of the noise may be irregular, which may contribute to the tracking marker, i.e., the peak of the second derivative of the RPOPL differences of the microsphere center pixel, in CDHM. Therefore, accurate positioning of the microsphere center is a crucial way to improve the SNR.

In conventional DHM, the imaging center is resolved by an image processing method, such as the Hough transform, and it is difficult to find the imaging centers of two or more adhered microspheres with overlapped diffraction patterns accurately. Therefore, accurate positioning of the centers is the key to measuring the axial displacement of microspheres with overlapped patterns.

In this paper, a CDHM-based reconstruction centering method (RCM) for displacement measurements of microspheres is proposed. Our aim was to suppress measurement noise through accurate centroiding. The advantageous effect of the RCM applied in CDHM was analyzed and evaluated by simulating ray tracing transmitted through microspheres and verified by motion experiments of microspheres in water. The testing results were with an axial resolution of 2 nm (out-of-focus microsphere)/4 nm (nearly in-focus microsphere) and an improved SNR of around 30%. In addition, the method was successfully applied to axial displacement measurements of microspheres in overlapped image patterns with a resolution of 2 nm.

## 2. Methodology

### 2.1. Analysis and Simulation

CDHM is set up with an off-axis digital holographic microscope. Both off-axis and in-line digital holograms can be resolved in this setup. Either the off-axis or in-line digital hologram is selected for reconstruction to calculate the displacement of the microsphere along the axis. The decision whether to reconstruct the off-axis or in-line digital hologram is based on an assessment of the severity of the impact on the twin images. This assessment is characterized by the critical reconstruction distance (CRD) boundary parameter.

In CDHM, if the reconstruction distance is less than the CRD, the ring pixels of the optical path length (RPOPL) method are implemented. The optical path difference (OPD) around the center and ring pixels is a maximum as compared to the other pixels. Therefore, the most critical step of RPOPL is to position the center pixel and resolve the positioning mark along the optical axis, i.e., the second derivative of OPD between the center pixel and ring pixels.

Previously, the CDHM method was built on the hypothesis that the rays incident through the microsphere are parallel to the optical axis and perpendicular to the imaging plane. In reality, however, the rays do not conform to the hypothesis. To improve the measuring precision and SNR of CDHM, more precise ray tracing should be analyzed.

By experimental results, it is found that the pixel point with the maximum phase, i.e., the maximum optical path length (MOPD), is not the imaging center of the microsphere. Since the ray transmitted through the center of the microsphere has the maximum optical path length (OPL) value, the central ray passing through the microsphere is not perpendicular to the imaging plane, and the incident rays are not parallel to the optical axis. Therefore, the optical path difference should be re-analyzed by ray tracing.

Unavoidable installation errors in the optical system should be the cause of non-uniformity in the imaging center and phase center. ① Mechanical installation errors cause the incident beam to be off-axis with the optical axis. ② The beams transmitted through the beam expander are spherical beams, not parallel beams.

**Figure 1:**
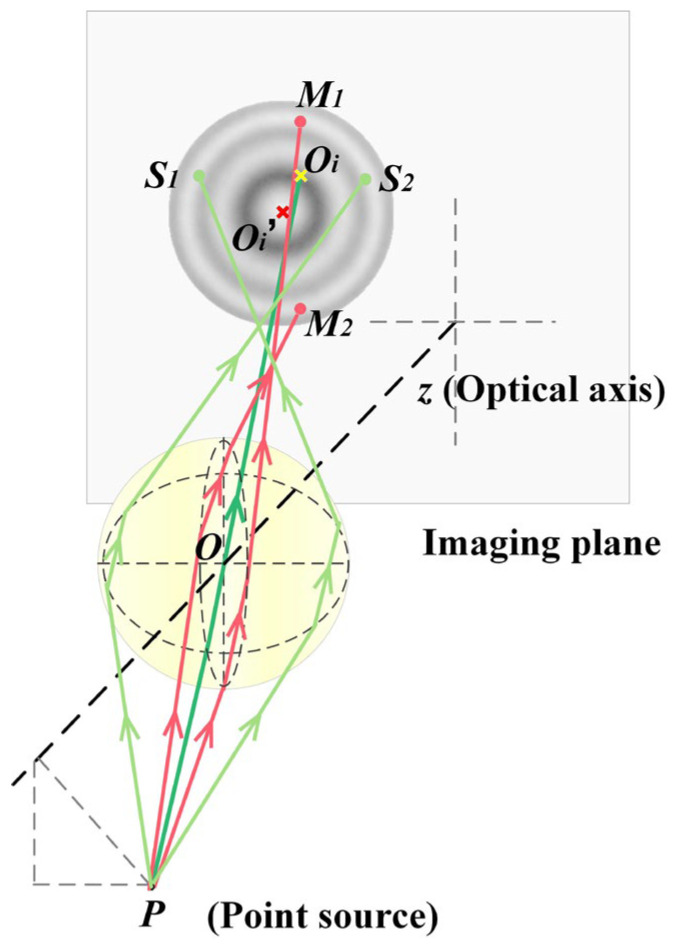
The beams transmitted through the microsphere.

Obliquely incident and non-parallel beams are equivalent to the divergent beams emitted by an off-axis point source. The beam emitted from an off-axis point source is neither symmetrical to the primary ray nor to the optical axis due to the presence of a Coma aberration, as shown in Figure 1.

**Figure 2:**
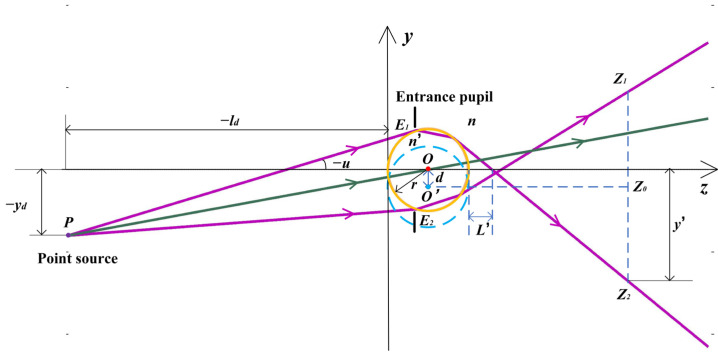
The marginal rays and central ray of the *PO_i_O_i_*′ plane in Figure 1.

In Figure 1, *P* is the point source introduced by the mechanical installation and beam expansion error; the Z axis refers to the optical axis, which is perpendicular to the imaging plane; *PS*_1_*S*_2_ is the sagittal plane; and *PM*_1_*M*_2_ represents the meridional plane. *PS*_1_, *PS*_2_*, PM*_1_, and *PM*_2_ are the marginal rays, while *PO_i_* is the central ray. The image of the microsphere at the imaging plane is formed by the projection of marginal rays. Hence, the imaging center *O_i_*′ is the *S*_1_*S*_2_*M*_1_*M*_2_ circle center. The marginal rays and the central ray in the *PO_i_O_i_*′ plane are shown in Figure 2.

Since there is no aperture diaphragm in front of the microsphere, the entrance pupil is in the microsphere. In Figure 2, *PE*_1_ is almost tangent to the microsphere. *E*_1_*E*_2_ is the entrance pupil, and *PE*_1_ and *PE*_2_ refer to the upper and lower marginal rays, respectively. At the imaging plane $z=Z_{0}$, *Z*_1_ and *Z*_2_ are at the image edge of the microsphere. In digital holographic reconstruction, the image is backpropagated at the distance of $Z_{0}-r$, where *r* is the radius of the microsphere. The Imaging center is at the midpoint of *Z*_1_*Z*_2_. In the reconstruction plane, *O*′ is the imaging center while *O* is the center of the microsphere through which the ray has a maximum OPL value.

To quantitatively evaluate the result caused by installation error, the RPOPL of the imaging center and reconstruction center were simulated by tracing rays through the microsphere.

According to the theory of optical path calculation for refractive spherical systems, the image intercept *L*′ is shown in Figure 2.

(1) $$L’=\frac{(L-r)\sin u}{\sin\left(u+2(1-n/n’)(L-r)\sin u/r+2\sum_{m=1}^{\infty }\frac{(2m-1)!!}{2^{m}m!(2m+1)}(1-{\left(\frac{n}{n’}\right)}^{2m+1}){\left((L-r)\sin u/r\right)}^{2m+1}\right)}-r$$

where *y_d_* is the field of the off-axis point source, *l_d_* represents the object distance from the point source to the first refractive sphere, *u* refers to the angular aperture of the rays emitted from the point source, *n* denotes the refractive index of the medium where the microsphere is immersed, and *n*’ is the refractive index of the microsphere. Figure 2 shows the variables of the upper marginal ray. The object intercept of the incident ray is *L.*

(2) $$L=y_{d}/\tan u+l_{d}$$

The image height *y*′ at the imaging plane is

(3) $$\begin{array}{l} y’=-\tan\left(u+2(1-n/n’)(L-r)\sin u/r+2\sum_{m=1}^{\infty }\frac{(2m-1)!!}{2^{m}m!(2m+1)}(1-{\left(\frac{n}{n’}\right)}^{2m+1}){\left((L-r)\sin u/r\right)}^{2m+1}\right)\\ {}*\left(z-\frac{(L-r)\sin u}{\sin\left(u+2(1-n/n’)(L-r)\sin u/r+2\sum_{m=1}^{\infty }\frac{(2m-1)!!}{2^{m}m!(2m+1)}(1-{\left(\frac{n}{n’}\right)}^{2m+1}){\left((L-r)\sin u/r\right)}^{2m+1}\right)}\right) \end{array}$$

where *z* is the reconstruction distance in object space.

The *OPL* of the ray is

(4) $$\begin{array}{l} OPL(z,L,u,y_{d})=n[y_{d}/\sin u-(L-r)\cos u-2\sqrt{r^{2}\cos^{2}u-L^{2}\sin^{2}u+2Lr\sin^{2}u}\\ +(L-r)\sin u\cot u_{2}’+(z-L’-r)/\cos u_{2}’]+2\sqrt{n{’}^{2}r^{2}-{\left[n(L-r)\sin u\right]}^{2}} \end{array}$$

where

(5) $$u_{2}’=u+2(1-n/n’)(L-r)\sin u/r+2\sum_{m=1}^{\infty }\frac{(2m-1)!!}{2^{m}m!(2m+1)}(1-{\left(\frac{n}{n’}\right)}^{2m+1}){\left((L-r)\sin u/r\right)}^{2m+1}$$

To calculate the difference in RPOPL, all pixels |x|=n&|y|≤n or |y|=n&|x|≤n form ring n. If the CCD has a pixel size of Δx×Δy, the RPOPL of ring n can be calculated by enumerations.

(6) $$\begin{array}{l} RPOPL_{n}(z)=\sum OPL_{p}(x,y,z)\\ \{x\in ((-n-1/2)\Delta x,(n+1/2)\Delta x]\;\&\;y\in ((n-1/2)\Delta y,(n+1/2)\Delta y],\\ x\in ((-n-1/2)\Delta x,(n+1/2)\Delta x]\;\&\;y\in (-(n-1/2)\Delta y,-(n+1/2)\Delta y],\\ x\in (-(n-1/2)\Delta x,-(n+1/2)\Delta x\;\&\;y\in (-(n-1/2)\Delta y,(n-1/2)\Delta y]],\\ x\in ((n-1/2)\Delta x,(n+1/2)\Delta x]\;\&\;y\in (-(n-1/2)\Delta y,(n-1/2)\Delta y]\} \end{array}$$

**Figure 3:**
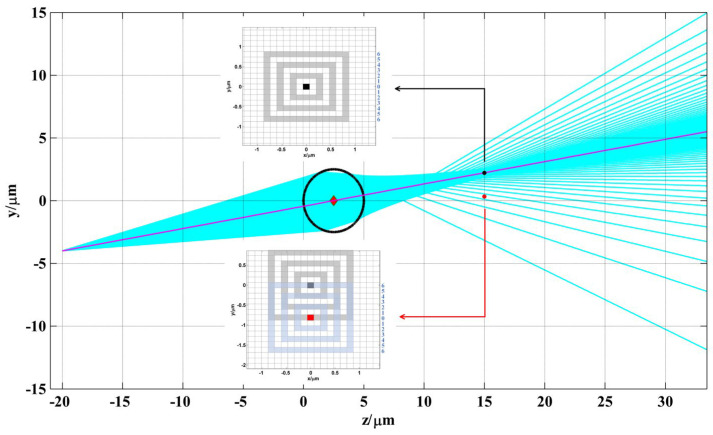
The rays tracing in sample microsphere.

The transmission of the microsphere is simulated in Figure 3. The diameter is 5 μm, which corresponds to that of silica, the refractive index of the microsphere is n=1.5, the refractive index of the water medium is n’=1.33, and the wavelength of the illuminating beam is λ=670nm. Suppose that $l_{d}=-20\;\mu m$, the field $y_{d}=-4\;\mu m$ can be obtained. To ensure the simulation was close enough to reality, the enumeration method was adopted to ensure that as many rays as possible were simulated. 10,000 rays were uniformly distributed in the entrance pupil and taken into simulation. Figure 3 shows the transmission of 100 rays.

**Figure 4:**
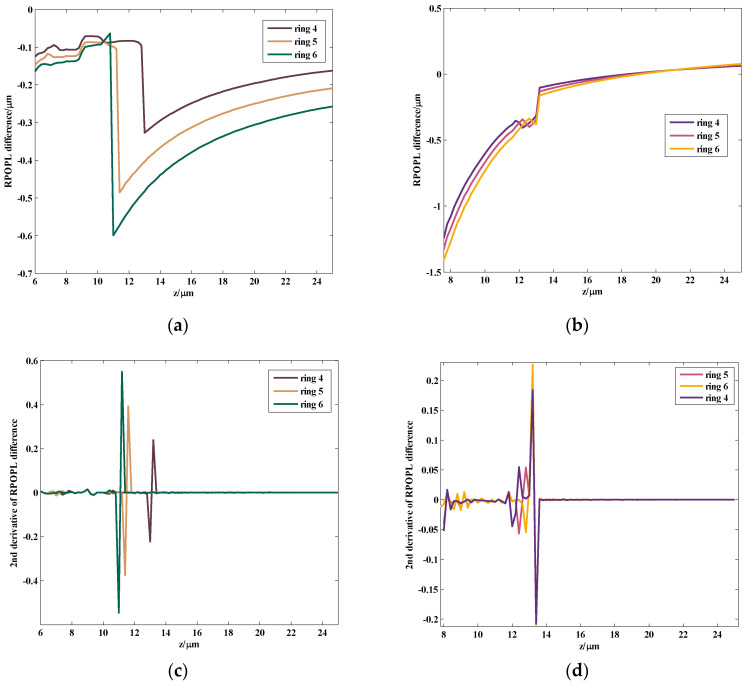
The RPOPL difference simulation of the reconstruction center pixel and the imaging center pixel. (**a**) RPOPL differences between various rings (ring 4, 5, 6, respectively) and reconstruction center pixel; (**b**) RPOPL differences between various rings (ring 4, 5, 6, respectively) and imaging center pixel; (**c**) second derivative of the RPOPL difference between the 4th, 5th, and 6th ring pixels and reconstruction center pixel; (**d**) second derivative of the RPOPL difference between the 4th, 5th and 6th ring pixels and imaging center pixel.

In Figure 3, at the imaging plane z=15 μm, the imaging center is the red point, while the reconstruction center through which the ray with the maximum OPL value passes is the black point. The pixel size of the CCD recorded in this study was 5.2 μm×5.2 μm, and the magnification was 54.77. Hence, the imaging center was at the midpoint of the marginal ray y=0.647 μm, while the black point was at y=0.222 μm. The distance between the two centers in the reconstructed plane was 4 pixels, when taking into account the magnification. The center pixel was numbered as 0, and other ring pixels were numbered according to the distance of the ring from the center pixel. The area of each pixel of the OPL on the reconstructed plane was calculated by Equations (3)–(5). The differences in the RPOPL of ring 4, ring 5, and ring 6, respectively, with the center pixel as the two centers mentioned above, are as shown in Figure 4.

As illustrated in Figure 4, the trends in RPOPL differences are similar for ring 4, ring 5, and ring 6. As shown in Figure 4a,b, the curve is sharper in the reconstruction center as compared to the imaging center. To characterize the grades of the bump, the second derivative of the RPOPL difference of the reconstruction center and imaging center is presented in Figure 4c and Figure 4d, respectively. The maximum peak-valley values of Figure 4c,d are 1.095 and 0.4367. In CDHM, the second derivative of the RPOPL difference is the effective marker to measure the axial displacement. The greater the peak-valley value of the tracking marker, the better the tracking marker will be identified. Using the reconstruction center as the center pixel point may increase the correct identification rate of the tracking marker by nearly 60%.

**Figure 5:**
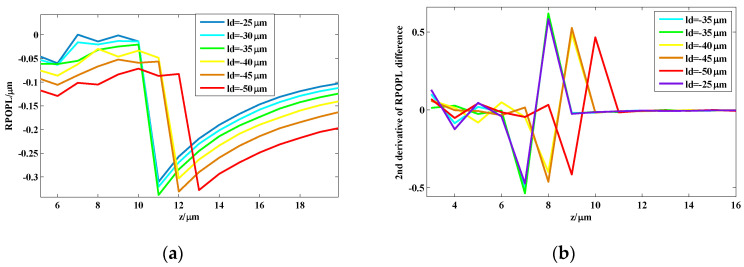
The RPOPL difference simulation of point sources at different locations (**a**) The RPOPL differences of the reconstruction center pixel; (**b**) second derivative of the RPOPL difference between the 6^th^ ring pixels and the reconstruction center pixel.

As is shown in Figure 5, point sources at different locations from $l_{d}=-50\;\mu m$ to $l_{d}=-30\;\mu m$ are simulated. The shapes of the RPOPL differences in the reconstruction center pixel and the second derivative of the RPOPL difference between the 6th ring pixels are similar to those of the point source located at $l_{d}=-20\;\mu m$. Therefore, using the reconstruction center pixel as the center pixel and resolving the second derivative of the RPOPL difference is an effective way to measure the axial displacement of the microsphere.

The *SNR* of a measurement is defined as

(7) $$SNR=10\mathrm{lg}\frac{\sum_{i=1}^{M}r{\left(i\right)}^{2}}{\sum_{i=1}^{M}{\left[r(i)-t(i)\right]}^{2}}$$

where r(i) is the measured result of axial displacement, t(i) is the moving distance of the microsphere, i.e., the moving distance of the piezo-stage.

### 2.2. Reconstruction Centering Method (RCM)

**Figure 6:**
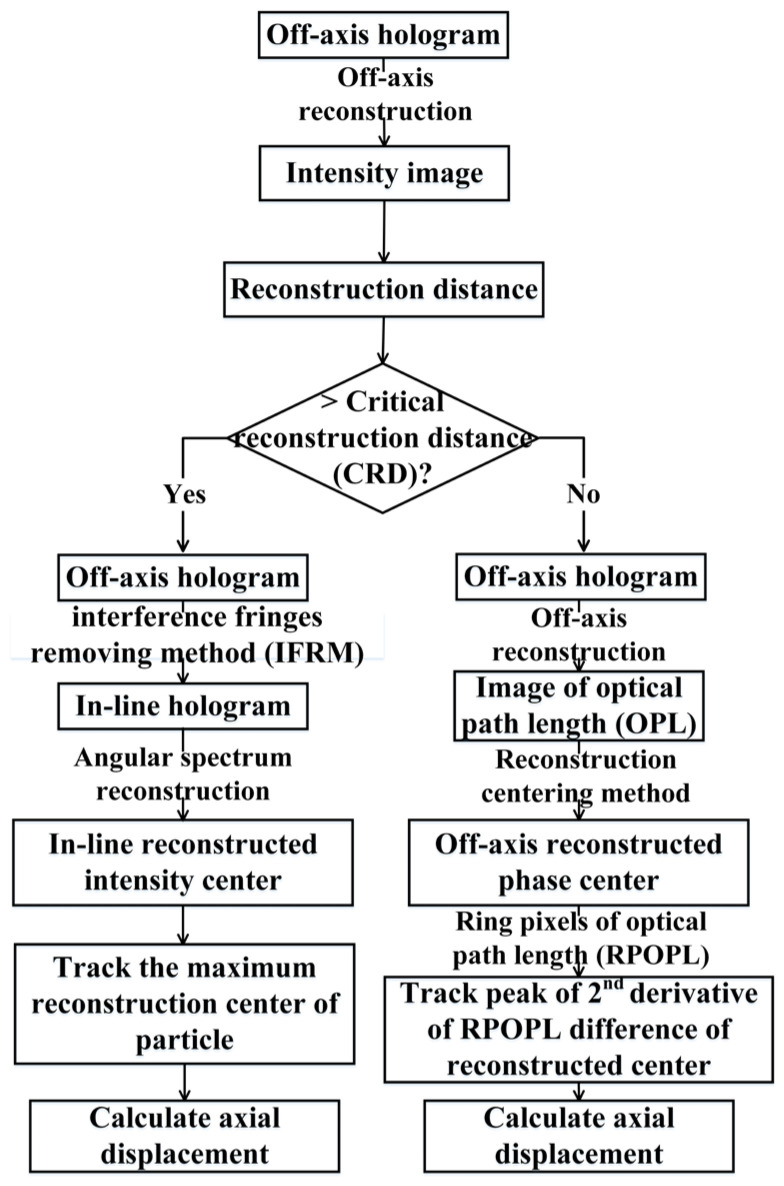
Diagram of CDHM combined with the RCM.

Based on the analysis above, the RCM was established to improve the effectiveness of the CDHM tracking marker. The flow diagram of this process is provided in Figure 6. The digital hologram is recorded by the digital image plane hologram and processed by the bidimensional empirical mode decomposition (BEMD) method to suppress the coherent noise [24,25,26].

The steps of CDHM combined with the RCM are as follows:

(1) Reconstruction of the off-axis hologram and comparison of reconstruction distance and CRD.

(2) If the reconstruction distance is less than or equal to CRD, the center of the reconstructed microsphere phase is used as the center pixel. The difference in RPOPL of the optical path length between the ring pixel and the center pixel is calculated. The peak of the second derivative of the RPOPL difference of the reconstructed center is traced, and the axial displacement is calculated.

(3) If the reconstruction distance is greater than CRD, an in-line hologram is obtained by the interference fringe removal method (IFRM). The center of the reconstructed intensity of the microsphere is considered the center pixel. The maximum intensity at the center of the reconstruction is traced along the optical axis, and the axial displacement is calculated.

### 2.3. Axial Displacement of Adhered Microspheres

The images of two or more adhered microspheres are overlapped. The center of overlapped diffraction rings is difficult to position using conventional image processing methods, such as the Hough transform method. Nevertheless, the reconstructed intensity or phase centers of adhered microspheres are apparently separated. Therefore, the RCM is especially suitable for measuring the axial displacement of adhered microspheres.

## 3. Apparatus and Experiments

### 3.1. Apparatus

**Figure 7:**
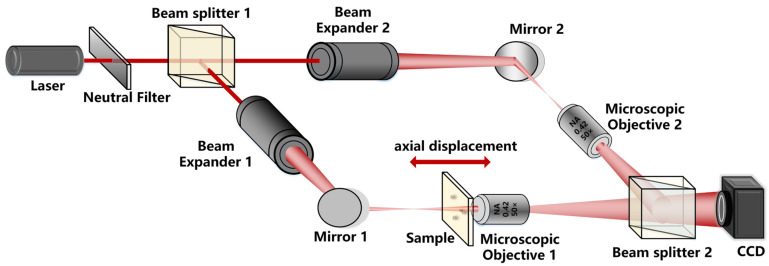
Experimental setup to measure displacement of microsphere movement.

The experimental setup utilized to measure the displacement of microspheres is the classical off-axis digital holographic microscope, which is shown in Figure 7. In this setup, the laser beam is emitted by a laser diode module (Thorlabs, Newton, NJ, USA, LDM670, λ=670nm). From the analysis in Section 2.1, the beams transmitted through a beam expander are spherical beams but not parallel beams. The information of the microsphere is then carried by the object beam, which is magnified by Microscopic Objective 1 (Mitutoyo, Houston, TX, USA, 50×, NA = 0.42).

### 3.2. Experiments on Sample Microspheres

The movement of the sample microspheres with different diameters (Polysciences, Warrington, PA, USA, silica with radius r=2.5 μm and r=5 μm, refractive index $n_{p}=1.5$; refractive index of the medium is $n_{l}=1.33$) was tested to evaluate CDHM combined with the RCM. The microspheres were fixed on a piezoelectric stage (Physik Instrumente, Irvine, CA, USA, S-303, 0.1 nm resolution), which moved along the optical axis during the experiment. The microspheres underwent 10, 5, 4, 3, 2 and 1 nm stepping processes along the *z* axis direction. We continuously took holograms while the piezoelectric stage was translating.

**Figure 8:**
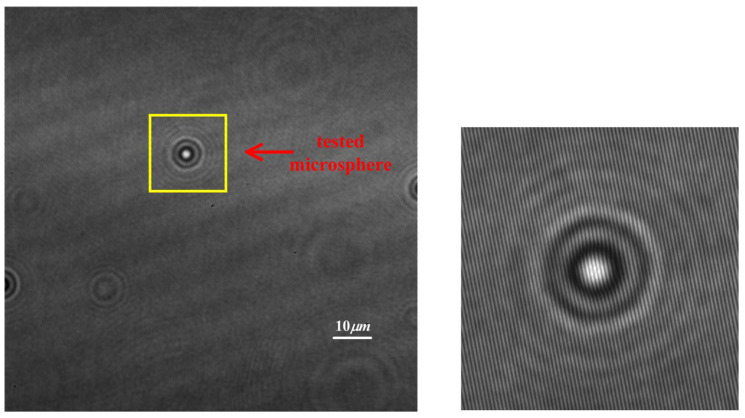
Off-axis digital hologram of an out-of-focus microsphere.

**Figure 9:**
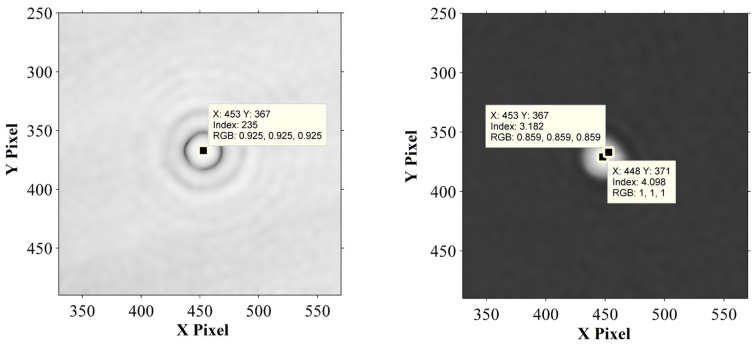
Hologram (the interference fringes are removed) and the reconstructed phase of the microsphere.

**Figure 10:**
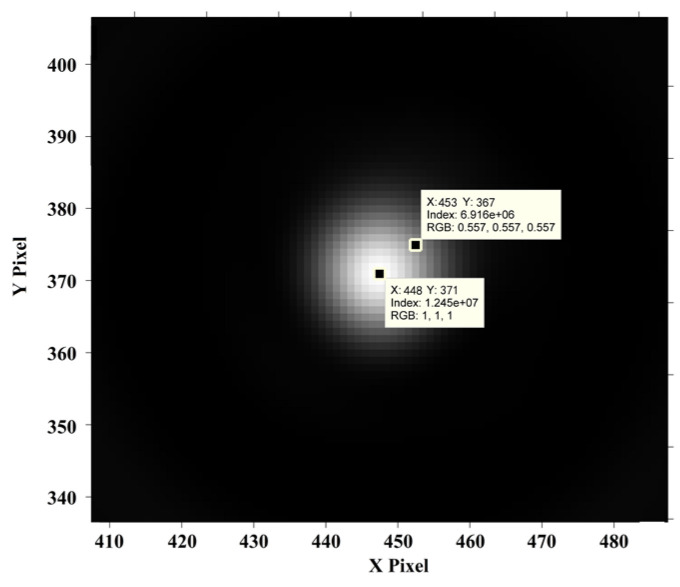
Reconstructed intensity of the hologram processed by IFRM.

The digital hologram of a microsphere with radius r=2.5 μm is shown in Figure 8. The reconstructed distance in image space is 63mm. The hologram (the interference fringes are removed) and the reconstructed phase of the microsphere are shown in Figure 9. The pixel point with the maximum phase, i.e., the maximum optical path length (MOPD), is located at (448, 371), while the imaging center is at (453, 367), proving that the central ray passing through the microsphere is not perpendicular to the imaging plane, and that the incident rays are not parallel to the optical axis. As is shown in Figure 9 and Figure 10, the reconstructed center is 6.4 pixels away from the imaging center.

From Figure 9 and Figure 10, the intensity ratio and phase ratio of the reconstruction center to the imaging center are 1.8 and 1.3, respectively. Since the reconstruction distance is greater than the critical reconstruction distance (CRD), the interference fringes are removed by IFRM. The in-line reconstruction is chosen to track the maximum intensity of the microsphere. The intensity variation of the reconstruction center and the imaging center are both resolved to verify the superiority of CDHM combined with the RCM compared to traditional CDHM.

**Figure 11:**
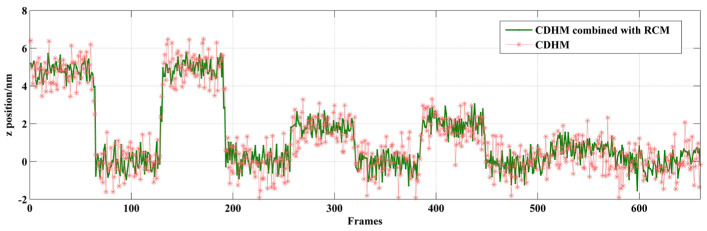
Displacement along the optical axis of an out-of-focus microsphere.

The microsphere is moved by ±5 nm, ±2 nm, and ±1 nm. The result of the displacement measurement is shown in Figure 11 and Table 1. As the piezo-stage moves, the digital holograms are recorded. As recording time passes, the microspheres are moved with the piezo-stage. The digital holograms of microspheres in different positions are recorded at different frames. In Figure 11, step jumping of positions with 5 nm and 2 nm can be clearly seen. But 1 nm step jumping cannot be judged. Therefore, the displacement measuring resolution of the out-of-focus microsphere is 2 nm. The data are shown in Table 1. The standard deviation of CDHM combined with the RCM is less than that of traditional CDHM. The SNR of both methods is resolved as 3.942 and 3.052 for CDHM combined with the RCM and traditional CDHM, respectively. Therefore, the SNR of CDHM combined with the RCM increased by 29.1%. The displacement measuring resolution of the out-of-focus microsphere is 2 nm.

**Figure 12:**
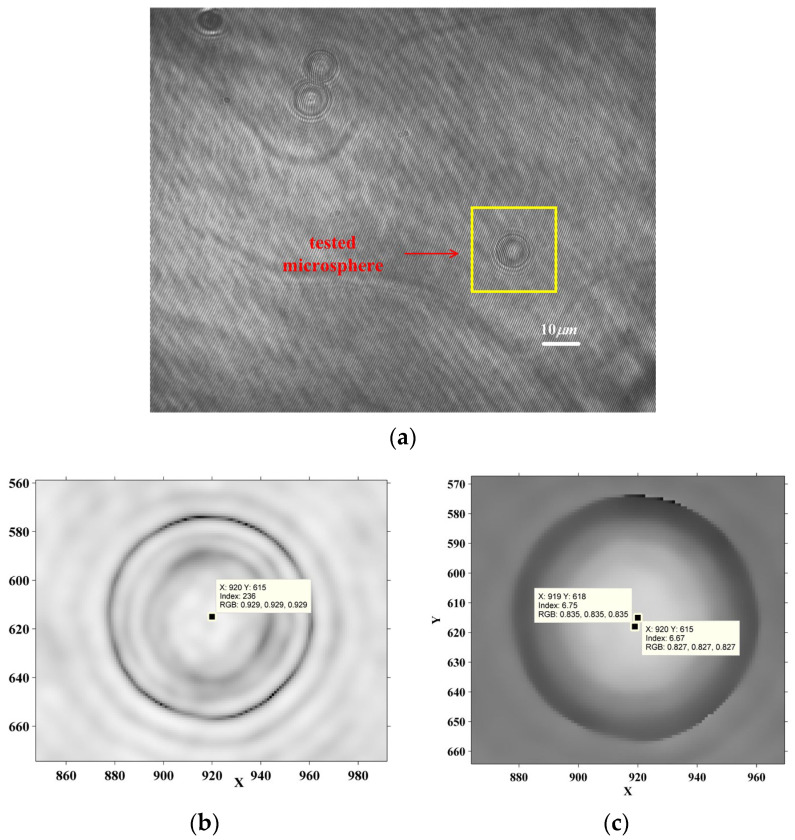
Off-axis hologram of a nearly-in-focus microsphere. (**a**) Digital hologram; (**b**) hologram without the interference fringes; (**c**) phase of microsphere.

**Figure 13:**
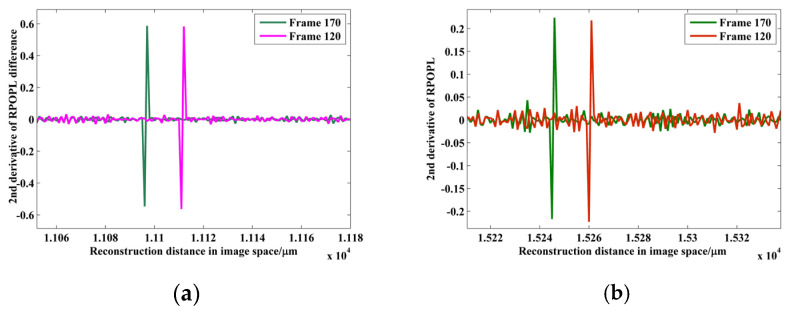
Second derivative of RPOPL difference between 6th ring pixels. (**a**) Reconstructed center pixel; (**b**) imaging center pixel for frame 120 and frame 170.

**Figure 14:**
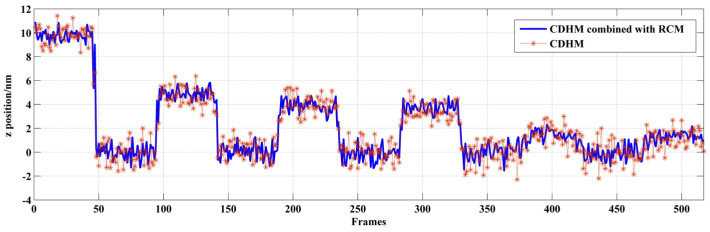
Displacement along the optical axis of a nearly-in-focus microsphere.

The digital hologram of a nearly in-focus microsphere is shown in Figure 12a. The diameter of the tested microsphere is 10 μm. The reconstructed phase is shown in Figure 12c. As is shown, the reconstructed center is 3.2 pixels away from the imaging center. The reconstruction distance is 8.7 mm, which is less than CRD. The off-axis hologram is reconstructed. RPOPL is utilized to resolve the displacement. The microsphere is moved by −10 nm, ±5 nm, ±4 nm, and ±3 nm. The second derivative of the RPOPL difference between the sixth ring pixels and the reconstructed center pixel for Frames 120 and 170 is shown in Figure 13a, while the RPOPL of the imaging center pixel is shown in Figure 13b. The maximum peak-valley value in Figure 13a is two times that in 13b, revealing that the tracking marker can be more accurately identified by the RCM. The result of the displacement measurement is shown in Figure 14 and Table 2. In Figure 14, step jumping of positions with 10 nm, 4 nm can be clearly seen. But 3 nm step jumping cannot be judged. Therefore, the displacement measuring resolution of a nearly-in-focus microsphere is 4 nm. The standard deviation of CDHM combined with the RCM is less than that of traditional CDHM. The SNRs of both methods are resolved as 10.79 and 8.13 for CDHM combined with the RCM and traditional CDHM, respectively. Therefore, the SNR of CDHM combined with the RCM increased by 32.7%. The displacement-measuring resolution of a nearly-in-focus microsphere is 4 nm.

**Figure 15:**
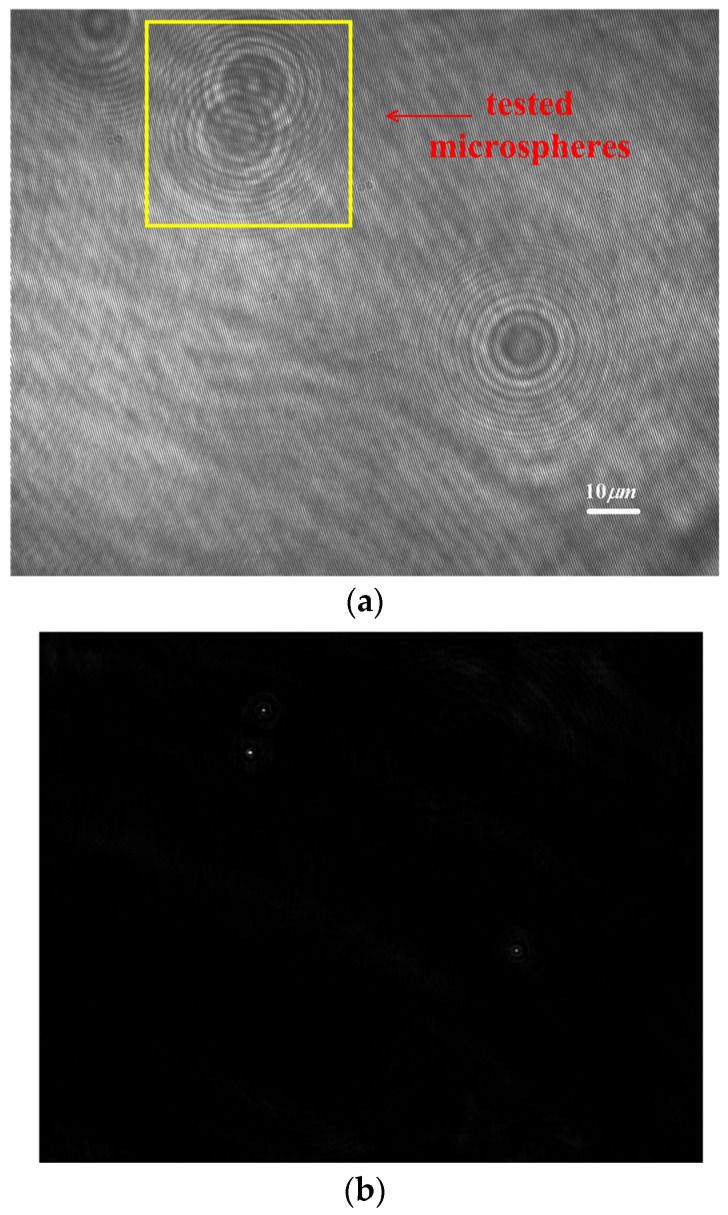
Digital hologram of overlapped microspheres. (**a**) Digital hologram; (**b**) reconstructed intensity.

**Figure 16:**
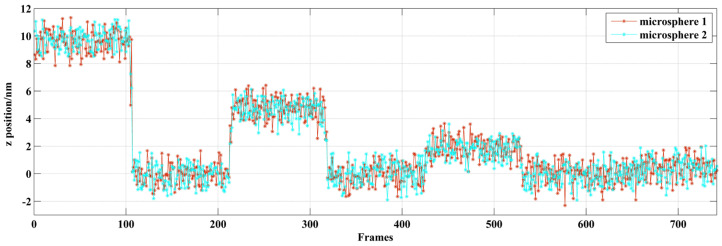
Displacement along the optical axis of overlapped microspheres.

CDHM with the RCM is applied to the measurement of overlapped microspheres. The digital hologram of two microspheres with overlapped patterns is recorded, as is shown in Figure 15a. The reconstructed distance is 56 mm. The intensity is reconstructed in Figure 15b. The overlapped microspheres are moved by −10 nm, ±5 nm, ±2 nm, and ±1 nm. The in-line reconstruction is utilized to resolve the intensity variation of the reconstructed center pixels. Since the reconstructed centers are separated, the tracking would not be influenced by the overlapped patterns. By applying CDHM combined with the RCM, the displacement result is shown in Figure 16 and Table 3. In Figure 16, the step jumping of positions with 10 nm, 5 nm, and 2 nm can be clearly seen. But 1 nm step jumping cannot be judged. Therefore, the displacement measuring resolution of overlapped microspheres is 2 nm.

### 3.3. Discussion

There are several other issues that need to be addressed when using the reconstruction centering method.

(1) There should be more than three rings to ensure the tracking marker is sufficiently visible. Therefore, the radius of the microsphere must have a length of at least 3 pixels in the CCD plane to achieve this. This limits the size of the microsphere being analyzed.

(2) The calculation speed of CDHM with the RCM should be improved. Deep learning, regional centering, and reconstruction are worth investigating in depth. The calculation time of CDHM with RCH is 14 frames/s (Windows 10, 64bit, 11th Gen Intel(R) Core (TM)i7-11800H @ 2.30GHz (16 CPUS), MATLAB R2012b).

## 4. Conclusions

In 3D motion measurements, measuring the axial displacement of a microsphere is relatively more difficult than the in-plane displacement. The phase and intensity of the microsphere center pixel are the keys to resolving axial displacement. In this study, a microsphere center position method called the RCM was investigated with a focus on improving measurement accuracy and suppressing noise. Through theoretical analysis and simulation, instead of regarding the imaging center directly as the microsphere center, the reconstructed center was proposed to be the microsphere center to suppress measurement noise and improve accuracy. The proposed method has several advantageous applications.

(1) Through analysis, the effective identification rate of the tracking markers will be improved by applying the RCM in CDHM. This theoretical argument is also verified by the SNR improvement of around 30% in experiments. Therefore, by utilizing the RCM, the immunity to noise of CDHM is improved.

(2) Overlapped microsphere patterns lead to a feature cross of rings that is hard to recognize by conventional methods. The findings of the experiments suggest that by applying CDHM combined with the RCM, the microsphere center can be positioned more accurately than the conventional method, especially for the overlapped microsphere. The proposed method can achieve an axial displacement resolution of 2 nm for both single microsphere measurements and overlapped microsphere measurements.

## Figures and Tables

**Table 1:** Displacement along the optical axis of an out-of-focus microsphere.

Frames	Result of CDHM/nm	Result of CDHM Combined with RCM/nm	Displacement of Stage/nm
2–62	4.74 ± 0.68	4.83 ± 0.38	−5
66–126	−0.01 ± 0.73	−0.06 ± 0.51	+5
130–190	5.02 ± 0.78	4.94 ± 0.43	−5
194–254	−0.09 ± 0.8	0.11 ± 0.49	+2
258–318	1.73 ± 0.72	1.82 ± 0.37	−2
322–382	−0.03 ± 0.81	−0.11 ± 0.49	+2
386–446	1.87 ± 0.71	2.04 ± 0.46	−2
450–510	−0.03 ± 0.7	−0.13 ± 0.47	+1
514–574	0.51 ± 0.75	0.75 ± 0.38	−1
578–638	−0.15 ± 0.69	−0.11 ± 0.51	+1
642–702	0.17 ± 0.78	0.2 ± 0.35	

**Table 2:** Displacement along the optical axis of a nearly-in-focus microsphere.

Frames	Result of CDHM/nm	Result of CDHM Combined with RCM/nm	Displacement of Stage/nm
2–45	9.82 ± 0.7	9.76 ± 0.43	−10
49–92	−0.14 ± 0.94	−0.03 ± 0.5	+5
96–139	4.69 ± 0.8	4.9 ± 0.47	−5
143–186	0.18 ± 0.78	0.04 ± 0.51	+4
190–233	4 ± 0.76	3.86 ± 0.56	−4
237–280	0.04 ± 0.84	−0.14 ± 0.52	+4
284–327	3.48 ± 0.68	3.72 ± 0.49	−4
331–374	−0.15 ± 0.92	0.03 ± 0.53	+3
378–421	1.45 ± 0.77	1.37 ± 0.5	−3
425–468	−0.16 ± 0.92	−0.1 ± 0.49	+3
472–515	1.16 ± 0.77	1.3 ± 0.41	

**Table 3:** Displacement along the optical axis of overlapped microspheres.

Frames	Result of Microsphere 1	Result of Microsphere 2	Displacement of Stage/nm
2–104	9.59 ± 0.8	9.76 ± 0.7	−10
108–210	−0.05 ± 0.72	−0.12 ± 0.76	+5
214–316	4.81 ± 0.78	4.66 ± 0.77	−5
320–422	−0.06 ± 0.73	0.02 ± 0.83	+2
426–528	1.88 ± 0.74	1.76 ± 0.68	−2
532–634	−0.02 ± 0.81	−0.17 ± 0.74	+1
638–740	0.38 ± 0.77	0.33 ± 0.78	

## Data Availability

The raw data supporting the conclusions of this article will be made available by the authors on request.
